# Epilepsy and the Administration of Bromide of Potassium

**Published:** 1865-07

**Authors:** G. Goddard Rogers

**Affiliations:** Physician to the West London Hospital, and late Medical Registrar to St. George’s Hospital; Grosvenor street, Grosvenor square


### EPILEPSY, AND THE ADMINISTRATION OF BROMIDE OF POTASSIUM.

By G. GODDARD ROGERS, M. D.,
*Physician to the West London Hospital, and late Medical Registrar to St. George's Hospital.*

A very interesting paper on the above subject, from the pen of Dr. M'Donnell, was published in the *Dublin Quarterly Review* of February last. It fell under my notice in May, when I was about to give a record of considerable success in the treatment of a case where, all other remedies having failed, I had been for a considerable time trying the bromide of potassium. After reading Dr. M'Donnell's observations, I determined to give the medicine a few months' longer trial before venturing to speak with any confidence as to its efficacy. To my own mind it is satisfactory that so long a time elapsed between the publication of Dr. M'Donnell's paper and my perusal of it. I came to the treatment of the patient mentioned below with an unbiased mind, and with a feeling that I had undertaken an almost hopeless task. I was, however, gratified by a measure of success, and strengthened in my belief that good results were to be obtained from bromide of potassium in certain forms of epilepsy, when my attention was directed to the above mentioned able article. I may just observe that I have given the drug to other epileptic female patients at the hospital with good effect, but the case I now append is the only one I have been able to watch for a prolonged term. Doubtless we are treading on soft ground if, for the present, we adopt Dr. M'Donnell's view, that "each case of epilepsy is in itself a study;" but I trust that ere long so many individual cases will have been observed of epilepsy connected with derangement of the female genital organs, as to enable us to form a group or groups of these affections amenable in a large percentage to a special remedy, even though we may in the present state of our knowledge only rudely conjecture its mode of action. With all deference, I beg leave to suggest that during a certain period—say from January, 1865, to March, inclusive—the various hospital physicians in this metropolis use the bromide of potassium in all cases of epilepsy where there is any reason for regarding uterine disorder as the source of the complaint. If no good result follow, yet no great harm can accrue, and at all events we shall have cleared away an untenable theory. There is true wisdom in the remark of M. Pouchet: "Every idea *à priori*, every hypothesis, is only good, if we accept it with a strong determination of abandoning it if the facts are no longer explicable by its means. Without this, its influence is disastrous."

*Case* 1. R. B——, aged thirty-four, married, with two children, the youngest eleven years old, first came under treatment at the West London Hospital in May, 1861. About six months after marriage she miscarried, and had for three weeks frequent attacks of syncope, followed a few weeks later by a convulsive seizure, during which she lost her consciousness. She does not remember being told afterwards whether she foamed at the mouth or bit her tongue. These attacks were few in number for some years; but she often had "fainting fits" and "sighing fits," during which she "felt lost"—(*petit mal?*)

In the early part of 1861 the fits recurred more frequently; so that she was obliged to give up her employment as occasional nurse. Feeling low spirited, and despairing of aiding her family, she came to the hospital. She was rather below the middle height, of florid complexion, with a wild staring expression, and very excitable manner. The heart's action was somewhat feeble, the breathing tranquil, and the bowels apt to be somewhat relaxed. Pressure of the crowd in the waiting room, or the anxious struggle to be amongst the first served with medicine after seeing the physician, would sometimes bring on a seizure. One I particularly remember was most violent, and lasted a long time. Although the true fits, or, as she always termed them, the "struggling fits," were principally confined to the pre-menstrual or post-menstrual epochs, yet they occasionally followed coition, as I learned from the husband, who, by the way, has been a noted pedestrian in sporting circles during the past twelve years.

I first gave turpentine and castor-oil draughts; but no worms were dislodged. Bichloride of mercury with bark was next tried; and remembering that valerian had been mentioned by some authorities as putting in a claim for approval, I tried it in many forms—infusion, ammoniated tincture, valerianate of iron, of zinc, and of quinine. Dr. Duncan Gibb, who was about that time my colleague, had been giving the bromide of ammonium to allay irritability and decrease sensibility of the larynx and pharynx prior to using the laryngoscope. Thinking the same drug might act beneficially on the mucous lining of the uterine organs, I administered it freely to my patient; but with no good effect. The same was the case with iodide of potassium; and I then accidentally refreshed my memory by reading Sir C. Locock's paper on the bromide.

From the 21st of November, 1862, to the 3rd December, R. B——— took bromide of potassium in four-grain doses three times a day, with compound tincture of valerian. Between these dates she had two fits of a mild character. The medicine was intermitted until the 9th, when she came in great distress, saying that she was suffering from severe flooding, and had had several violent fits, on one occasion nearly falling into the fire. She was ordered to resume the medicine.

Jan. 2nd, 1863.—To leave off the tincture of valerian, and take ten grains of the bromide in water three times a day.

8th.—Two severe attacks.

16th.—No seizure since last entry.

She now took quinine and ammonia up to March 6th. The seizures were very frequent during a portion of this time, and she suffered severely from menorrhagia.

From the 13th March to 12th May, she took ten grains of bromide three times a day, and much improved. In fact, she relied solely on the medicine, and declared that the fits would return directly she left it off. To test this statement, during the latter half of July, I merely prescribed a placebo of aromatic water; but so great was her distress that she implored me to allow her to resume the original medicine, and during August and September she took fifteen grains of the bromide three times a day. I afterwards increased the dose to a scruple. Beyond this dose I never found occasion to go with the patient, and, from my experience in other cases with larger doses, I am compelled to differ with Dr. M'Donnell when he says that ten grains three times a day is "too small a dose to develop any good result."

In January, 1864, I discharged my patient, she not having experienced a severe fit, or one of the *petit mal* description, for more than three months. She resumed her former work of nursing, and I saw nothing more of her until the 10th of June last, when she came to me complaining of vertigo and "fullness about the throat," the "choking aura," which was the sure precursor of a seizure at a menstrual period. Two days later the seizure occurred, but it was of a very mild type. I kept her on scruple doses of the bromide up to the 15th of July, seeing her during this time on six occasions. She remained quite free from attacks up to the time of her dismissal in July, and on the 3rd November, I heard that her good health had been uninterrupted. I do not mean to say that she is cured; but in 1861 and 1862, her life was a burden in consequence of frequent attacks, and from these she has enjoyed an immunity for three, four, or five months.

Systematic writers on materia medica have dwelt much on the anaphrodisiac influence of bromide of potassium. I do not think its beneficial influence is to be looked for in a simple lowering of the sexual power. When that power is unduly drawn upon, a degree of irritation is produced which the bromide may calm. But in the case above related, I had the fullest opportunities of learning from the husband that there never had been any diminution in his wife's *empressement.* And to arrive at greater certainty, the husband coming under my care for some trivial ailment during his wife's attendance at the hospital, I took the opportunity of administering the bromide of potassium to him in fifteen and twenty grain doses three times a day. The information supplied after the lapse of a month satisfied me that here, at all events, the depressing propriety of the drug was *nil.* Where there is irritation and frequent priapism, the result of onanism or venereal excess, and where there is every reason to conjecture that such irritation is at the bottom of the epileptic seizures, then the bromide of potassium is, I think, of service. Dr. M'Donnell has promised us some further observations, which it is probable will throw still more light on this important subject.

As confirmatory of the conjecture hazarded above respecting epilepsy in the male subject, I add a few notes of an hospital case, which will bring this paper to an end.

*Case* 2. E. B——, aged sixteen, came to the West London Hospital in June last. His appearance is heavy, he complains of loss of memory, is partially deaf, and the pupils are much dilated. At twelve years of age, when working in a gun factory, he contracted bad habits, which he has continued to indulge up to the present time. Sometimes he has practised self abuse three and four times a day. The bowels are costive, and there is a feeling of weight at the vertex. Before he was thirteen years of age he had several fits, which his friends describe to me as most severe, his struggles being extremely violent, and his tongue often bitten through. There was an intermission of eighteen months, followed by a fresh series of fits; and he then went to Salisbury Infirmary. The change of air and the medical treatment effected but little good; and when he returned to London he had "twitchings of the face, and fits almost every day. In June and July he was under my care, and I gave him the bromide three times a day in doses gradually increased to twenty-five grains. The attacks gradually became less frequent, and he was entirely free from them all through August. On the 2nd of September his friends brought him again, as he had suffered from a slight fit during the night. I resumed the bromide; and, after taking it a fortnight, he felt better, and had no "twitchings" and no sign of a fit. Up to the present date, (Nov. 21,) this lad has had no further seizures; and I think it reasonable to regard his improvement as due to the influence of the drug, coupled with obedience to my enjoining a strict abstinence from vicious habits.

Grosvenor street, Grosvenor square.

---

☞ Dr. E. L. Holmes, Chairman of the Committee on Ophthalmology, Illinois State Medical Society, has called our attention to the fact, that his remarks upon pressure in conjunctivitis, and the use of the muriate of ammonia in iritis, were not included in his Report, as stated in the published transactions of the recent meeting of the Society, but were made informally; full credit for their introduction into use in this country being accorded to Surgeon J. S. Hildreth, of the U. S. Ophthalmic Hospital, Chicago.

